# The development of a training course for clubfoot treatment in Africa: learning points for course development

**DOI:** 10.1186/s12909-018-1269-0

**Published:** 2018-07-13

**Authors:** Tracey Smythe, Grace Le, Rosalind Owen, Birhanu Ayana, Linda Hansen, Christopher Lavy

**Affiliations:** 1 International Centre for Evidence in Disability, London School of Hygiene & Tropical Medicine, Keppel Street, London, WC1E7HT UK; 2 Nuffield Department of Orthopaedics, Rheumatology and Musculoskeletal Sciences, University of Oxford, Oxford, UK; 3 Global Clubfoot Initiative, London, UK; 4 Black Lion Hospital, Addis Ababa, Ethiopia; 5 CURE International, Beit CURE Hospital, Lusaka, Zambia

**Keywords:** Clubfoot, Congenital talipes equinovarus, Ponseti, Africa, Training, Clinical skill, Course development

## Abstract

**Background:**

Clubfoot is a common congenital musculoskeletal disorder that causes mobility impairment. There is a lack of trained mid-level personnel to provide clubfoot treatment in Africa and there is no standard training course. This prospective study describes the collaborative and participatory approach to the development of a training course for the treatment of clubfoot in children in resource constrained settings.

**Methods:**

We used a systems approach to evaluate the development of the training course.

Inputs: The research strategy included a review of context and available training materials, and the collection of data on current training practices. Semi-structured interviews were conducted with seven expert clubfoot trainers. A survey of 32 international and regional trainers was undertaken to inform practical issues. The data were used to develop a framework for training with advice from two technical groups, consisting of regional and international stakeholders and experts.

Process: A consensus approach was undertaken during workshops, meetings and the sharing of documents. The design process for the training materials took twenty-four months and was iterative. The training materials were piloted nine times between September 2015 and February 2017. Processes and materials were reviewed and adapted according to feedback after each pilot.

**Results:**

Fifty-one regional trainers from Africa (18 countries), 21 international experts (11 countries), 113 local providers of clubfoot treatment (Ethiopia, Rwanda and Kenya) and local organising teams were involved in developing the curriculum and pilot testing. The diversity of the two technical advisory groups allowed a wide range of contributions to the collaboration.

Output: The resulting curriculum and content comprised a two day basic training and a two day advanced course. The basic course utilised adult learning techniques for training novice providers in the treatment of idiopathic clubfoot in children under two years old. The advanced course builds on these principles.

**Conclusion:**

Formative research that included mixed methods (both qualitative and quantitative) was important in the development of an appropriate training course. The process documentation from this study provides useful information to assist planning of medical training programmes and may serve as a model for the development of other courses.

**Electronic supplementary material:**

The online version of this article (10.1186/s12909-018-1269-0) contains supplementary material, which is available to authorized users.

## Background

Clubfoot, or congenital talipes equinovarus, is a common congenital disorder that causes mobility impairment if untreated. It is a structural and functional deformity where the child is born with the foot turned inwards [1]. The Ponseti method [2, 3] is promoted as an effective and low cost treatment of clubfoot [4]. This minimally invasive method includes a correction phase and a maintenance phase, and can be delivered by trained mid-level health care providers in resource constrained settings [5]. The correction phase of treatment involves the simultaneous correction of three components of the clubfoot deformity, with the equinus (downward pointing of the foot) corrected last. The manipulated foot is held in a series of long leg (toe to groin) plaster of paris casts, with the knee at 90 degrees. Manipulation and casting usually occurs weekly. The plaster cast retains the degree of correction and allows the soft tissue time to remodel. Once the cavus, adductus and varus have been corrected, an outpatient procedure to cut the Achilles tendon (heel tendon), known as a tenotomy, is usually needed to correct the rigid downward position of the foot. The final cast remains for 3 weeks to allow the Achilles tendon to re-grow in this lengthened position. To prevent recurrence of the deformity, it is recommended that a foot abduction brace is worn for 23 h/day for 3 months following correction, and then at night until the child is 4 years old [6].

Despite the worldwide spread of the Ponseti method for clubfoot [7], there is a discrepancy between what is known about effective clubfoot management and its availability to children with clubfoot in Africa. A major barrier is trained health care providers. The World Health Organization (WHO) recognises a health workforce crisis in Africa [8] and the need to share resources to narrow the gap between the ideal health workforce and the capacity of training institutions [9].

The Africa Clubfoot Training (ACT) project was developed in response to requests to build local training capacity in the Africa region and to develop training materials with standard elements for novice clubfoot treatment providers. The project aimed to align with local priorities though joint planning and co-ordination, and to establish a long-lasting partnership and community of practice. It proposed to:

i. Develop a simplified training course for novice clubfoot treatment providers with standard elements
ii. Strengthen training and delivery capacity for clubfoot treatment in sub-Saharan Africa through providing national clubfoot trainers with standardised and evaluated training materials
iii. Build capacity for clubfoot training and mentoring through developing an integrated training of trainers course

Historically a narrow technical focus without contextual understanding and limited teamwork [9, 10] have led to a lack of agreement on curricula and relevant frameworks for medical education. The tendency of professionals to act in isolation compounds this difficulty [9]. While frameworks for successful partnership networks and principles of good collaboration are reported [10–14], there is a paucity of data to inform dialogue on the development of medical training [15]. Many training courses include components of knowledge, skill and competency [16], however formative research used to inform the design of content is rarely reported [17]. Formative research is systematic and process oriented. It allows the collection of data (both qualitative and quantitative) and analysis of what elements of the training work, what needs to be improved and how this process may occur [18]. It aims to develop and improve training design from an early stage when opportunities for influence are likely to be greatest, and it allows improved understanding of the factors that influence training [19]*.*

There is little evidence of the effectiveness of training and mentoring health care providers in the management of clubfoot in Africa, or how such interventions can be best designed to be feasible and appropriate. This prospective study describes a systems approach [20] to the design and implementation of a health related training course. We describe the inputs and processes [21] that the ACT project used to create the training course, and how these were achieved. The lessons learned may be useful for development and implementation of other medical training courses.

## Methods

### Inputs

#### Partnership of stakeholders

The ACT project is a partnership between the Nuffield Department of Orthopaedics, Rheumatology and Musculoskeletal Sciences (NDORMS) at the University of Oxford, the Global Clubfoot Initiative (GCI), CURE Clubfoot and CURE Ethiopia Children’s Hospital, in co-ordination with the Ministries of Health in Ethiopia and Rwanda. These partners formed a core project team and informed project planning and course design through comprehensive knowledge of regional context and needs. The Tropical Health Education Trust (THET) funded the ACT project through the Health Partnership Scheme funded by the UK Department for International Development. The London School of Hygiene & Tropical Medicine provided monitoring and evaluation expertise.

#### Comprehensive needs analysis

A situational needs analysis and scoping meeting was undertaken in March 2015 with the core project team. The current understanding, context and needs for a training course were mapped extensively.

#### Review of existing training materials and current practice

Initially, a review of available training materials, teaching methods and processes was undertaken. Two researchers in Public Health then conducted semi-structured interviews with regional and international trainers in the Ponseti method of clubfoot management to collect data on current training practices and challenges. The interviews were transcribed and coded to identify common themes. Following the interviews, an online survey was created and regional and international trainers, purposively selected as experts in the field, were invited to participate. The survey was designed to identify which training practices provided desired clubfoot management behaviours and potential areas for improvement. It aimed to understand practical issues, such as whom to include in the training, current knowledge and skills gaps, and follow up mentoring requirements (Additional file 1). Throughout this review process, published materials and current literature were explored [22] to identify best practice in both clubfoot management and principles of adult learning (for example, providing effective feedback) [23].

#### Expert consultation

A wide spectrum of experts were involved in the decisions of what should be included in the training and how this should be achieved. The experts were invited to participate in one of two technical advisory groups (TAG). The Africa TAG consisted of trainers in clubfoot management (Ponseti technique) throughout Africa and experts from non-governmental organisations (NGOs) that currently support clubfoot management in Africa; representatives from CBM, CURE, International Committee of the Red Cross (ICRC), MiracleFeet, Mobility Outreach International (MOI) and Ponseti International Association (PIA) were invited to participate. The UK TAG consisted of members of the UK Clubfoot Consensus Group [24] and medical educationalists. The roles of each TAG were outlined at the beginning of the project and included the review of course material for appropriate training delivery, technical and methodological accuracy, and suitability for delivery in a low-income setting.

#### Training of trainers

Regional trainers were identified to deliver and pilot the ‘Basic Provider Course’ (BPC) training and supervise new providers. The trainers were chosen based on willingness to participate, knowledge of the topic and their strategic role as key clubfoot trainers in their country. They included orthopaedic surgeons, physiotherapists, physician assistants, prosthetists and orthotists, nurses and orthopaedic technicians. The mean length of time that the trainers had used the Ponseti method was 7.8 years (95%CI 6.5–9.0) and the average number of trainings delivered was 6.4 (95%CI 4.4–8.4). The regional trainers attended a two-day, customised “Train the Trainer” (TTT) course delivered by clubfoot experts and immediately delivered the two-day ‘Basic Provider Course’ (BPC) themselves, to reinforce newly learned principles and to give feedback on the BPC structure and content.

#### Equipment

Rubber models and skeleton models were required. A list of material inputs (e.g. plaster of paris, underwrap, tenotomy kits, buckets) was created over the pilot trainings to assist future training courses and is outlined in Additional file 2.

#### Patients

Children with clubfoot, under the age of 6 months, were assessed and treated by providers under close supervision. All caregivers were read an information sheet about the training and given an opportunity to ask questions. If they agreed to participate, written consent was taken. The caregiver was required to provide written consent and to remain present throughout the assessment as per national requirements. Transport costs were reimbursed and referral services available were mapped pre-emptively to ensure appropriate onward referral for any children that required further intervention.

### Process

#### Plan: Partnership development plan, workshops, meetings, draft documents

**Fig. 1:**
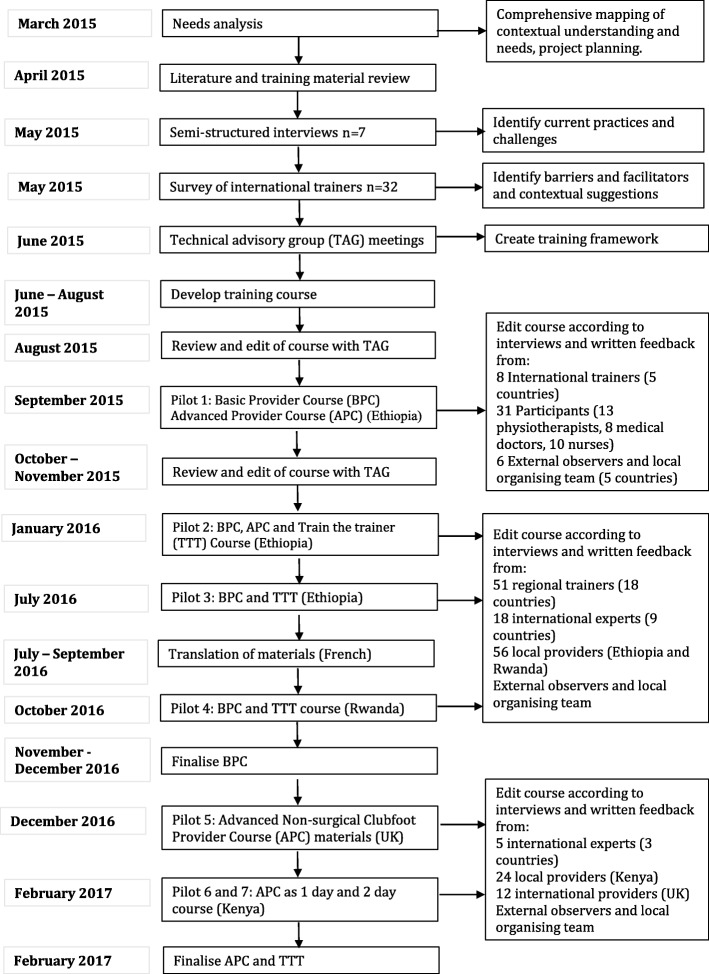
Timescale of key activities in the development of the training course

All partners that formed the core project team formally committed to a partnership development plan at the beginning of the project. The BPC and APC training goals and design were developed from the extensive needs analysis, project planning, literature search, interview and survey data. They were presented at web meetings with the two TAGs and subsequently commented on over a two-week period. The goals and design were modified accordingly and topics where consensus was not reached were noted. Meetings occurred after each pilot with the core project team and draft documents were shared electronically for further comments and suggestions, after which they were amended. This occurred seven times. The APC was presented and discussed with regional trainers on the final day of two BPC pilots. Figure 1 outlines the timescale of key activities.

#### Design: consensus approach

A list of topics that required consensus was generated where evidence in the literature was lacking or unclear. Two additional web meetings were held with the TAGs prior to the delivery of the pilot training to discuss the topics where consensus was required. As the aim for the BPC was to create a course that was simple to understand for novice providers, materials with a standardized approach on the controversial areas in clubfoot treatment were required. Further consensus forums were therefore scheduled in the pilots for discussion with regional trainers.

#### Development: iterative approach

The BPC content and structure was edited twice before the first pilot in September 2015. The training material was piloted four times. Semi-structured interviews with trainers and trainees were conducted during each pilot training and collated with feedback from verbal debrief sessions and written questionnaires. The training materials were reviewed and adapted according to feedback after each pilot. This process evaluation allowed further development of the training material and substantial changes were made to the timing and content of the training course. The comprehensive needs analysis allowed a good foundation for the format of the course, however at the beginning of the project partnership, the precise content of the training course was unknown. Through the iterative approach, it was identified precisely where novice providers required support after basic training and the APC content was developed into a 2 day course from the recommendations made at the end of the BPC. The process evaluation for the five APC pilots was the same as for the BPC.

#### Delivery

The two-day BPC was piloted with eight international trainers in September 2015. The content, materials, supervisory and mentorship structure [16] of the BPC was then piloted with 51 regional trainers in Ethiopia (2 courses) and Rwanda (1 course) and included both Anglophone and Francophone trainers. The one-day APC was piloted in Ethiopia (2 courses) and the UK (1 course) and a one-day and two-day version of the APC course was piloted in Kenya.

#### Monitoring

**Fig. 2:**
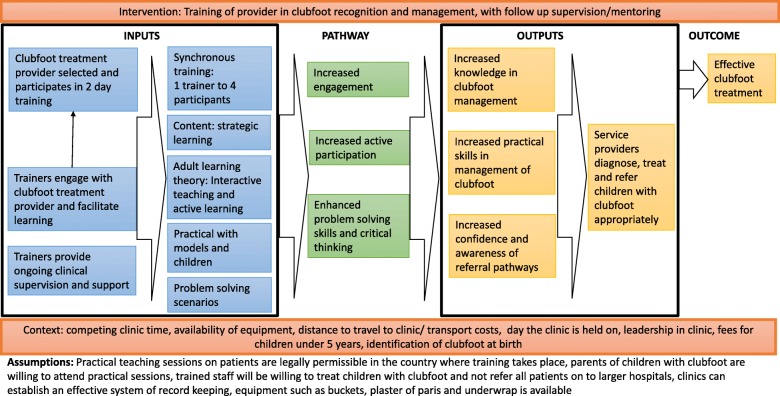
Theory of change for the development of the BPC training course

A theory of change (ToC) was developed to hypothesise the pathways that may contribute to behaviour change in the BPC training (Fig. 2). Regional trainers attending the TTT course completed a knowledge MCQ and confidence matrices immediately pre- and post-course, and a further confidence matrix after applying teaching skills in delivering a BPC.

The ToC was adjusted throughout the twenty-four months as further assumptions and contextual factors were considered. The standard elements to be delivered in training were defined in order to inform a skills checklist, knowledge and confidence pre- and post-training. Quantitative data were collected through pre- and post-training questionnaires to monitor efficacy of training and to identify areas for improvement and increased learning opportunities. Knowledge was assessed before and after the course with a pilot tested single best answer multiple-choice questionnaire (Additional file 3). Long-term knowledge retention was not assessed. Confidence in key skills in clubfoot management was assessed before and after the course with a pilot tested confidence matrix. Extensive written and verbal feedback was obtained from national, regional and international trainers following each pilot to identify areas for improvement.

#### Ethics, consent and permissions

The London School of Hygiene & Tropical Medicine granted ethical approval for this study ref.:10412 /RR/3466. Informed written consent was obtained from all participants.

## Results

### Training goals

The design process for the curriculum and content of the training course for clubfoot treatment in Africa took twenty-four months (March 2015 – February 2017) and was iterative. The comprehensive needs analysis proposed a project design that included goals to:

i. Create and pilot a simplified basic course with key messages and increased practical opportunities and videos, with the use of a standardised teaching method to promote critical thinking and problem solving
ii. Create and pilot an advanced course and an integrated training of trainers
iii. Pilot the test materials with trainers from throughout Africa to ensure input to material development and enhance acceptability, feasibility and ownership
iv. Ensure manuals include logistics for administration staff and briefings for the trainers to promote the link between training and follow up supervision and mentoring.
v. Purposively select pilot locations and regional trainers based on extensive knowledge and capacity
vi. Translate the materials into French due to needs of the region
vii. Integrate principles of adult learning.

### Participation

Of the 26 international and regional trainers invited to the first interviews, seven (27%) were available to participate. Participants reported the need for training materials with consistent language and messages, and understandable learning outcomes. The importance of practical components in training and follow up mentoring were highlighted, as was the need to facilitate expectations of what practical skills a provider may develop through participation in the training programme.

Thirty-two of 100 trainers (32%) responded to the online survey. Collectively, the 32 trainers had held courses in 54 countries. Twenty of the 32 trainers (63%) had delivered more than 10 courses. Seventy percent had used their own materials for training and 63% had used materials from the Global Clubfoot Initiative (GCI). Eighty-three percent advised on a separate basic and advanced provider course. The need for refresher training and mentoring was a key theme, in addition to requests for targeted training at an appropriate level for novice clubfoot providers. The creation of opportunities to share the previously developed curricula, best practices and lessons learned were common goals.

### Output, basic provider course

The resulting BPC has a focus on adult learning techniques for the training of novice providers in the treatment of idiopathic clubfoot in children less than 2 years of age. The final curriculum and content of the BPC focuses on a problem solving approach with group work and practical exercises to promote optimal learning and facilitate active engagement. It is designed to be delivered face to face over 2 days, or as fourteen stand-alone modules, with a sequence of learning tasks that progress from simple to more complex. If delivered as stand-alone modules, this would be in response to a specific training need or contextual issues that were identified by a clinic or individual. It contains clearly defined content and learning objectives for the development of required skills for the novice provider (Additional file 4 outlines the training courses). The structure of the BPC allows for the teaching of content through small group and other interactive activities and communication is promoted through built-in open questions to stimulate dialogue. The learning outcomes for novice local providers were based on the diversity of cadre to be trained and the need for a training course with standard elements.

### Learning outcomes BPC

The BPC was therefore designed with three main learning outcomes; providers of the Ponseti method of clubfoot treatment will be able to:

i. Explain the clubfoot deformity and the method of correction using the Ponseti technique;
ii. Demonstrate practical skills in treating a child under 2 years presenting with idiopathic, previously untreated clubfoot; and
iii. Identify more complex cases, when treatment is not progressing as would be expected and when to seek guidance with these cases.

### Output, advanced provider course

The APC was designed to build on the knowledge, competence and opportunities for mentoring that were developed through the BPC. The main objectives include:

i. To refresh understanding and skills in basic Ponseti treatment, and to add advanced knowledge to these
ii. To develop understanding and skills in non-surgical management of challenging cases such as atypical, recurrent, neglected, and secondary clubfoot
iii. To facilitate exchange of knowledge through case discussion of challenging cases
iv. To establish a common approach to measuring and improving quality of care in clinics, to encourage reflection on what is and what is not working well in own practice / clinic setting, and to identify priority actions
v. To promote consideration of how parents can be supported to promote treatment adherence during clubfoot treatment.

### Application of formative research

Consensus topics included, but were not limited to, the definition of recurrence of the clubfoot deformity, the definition of complex and atypical clubfoot, Pirani score and positioning, and the brace review protocol. Consensus on the format, content and structure was gained after the fourth pilot course. The need for a third training course that includes surgical management was identified.

As planning progressed, new questions were identified. Feedback included comments on individual slides, clarity and context of images, organisation and order of topics, consistency of language, context specific details, level of difficulty, and the identification of further practical opportunities.

The diversity of advisory groups and trainers allowed a wide range of contributions to the collaboration, but also complexity in the management of different interests. Table 1 outlines the results of the demographic analysis of the pilot courses.

**Table 1:** Demographics of the pilot courses

Pilot number	1	2	3	4	5	6	7	Total^a^
City, Country	Addis Ababa, Ethiopia	Addis Ababa, Ethiopia	Addis Ababa, Ethiopia	Kigali, Rwanda	London, UK (1 day)	Nairobi, Kenya (1 day)	Nairobi, Kenya (2 day)	
Dates of pilot trainings	23–24 September 2015	25–29 January 2016	25–29 July 2016	24–28 October 2016	2 December 2017	26 January 2017	24–25 January 2017	21 days
Type of pilot training	BPC and APC	BPC, APC and TTT	BPC and TTT	BPC and TTT	APC	APC	APC	
Number of International experts delivering training	8	9	9	9	5	5	5	21
Countries represented by International experts	Ethiopia, Norway, Netherlands, UK, Zimbabwe	Australia, Ethiopia, UK, Zimbabwe	Australia, Ethiopia, Kenya, Tanzania, UK, Zimbabwe	Australia, Canada, Cameroon, DRC, Rwanda, Switzerland, UK, Zimbabwe	UK	UK, Kenya, Australia	UK, Kenya, Australia	13
Number of organisers for training course	4	3	3	3	2	3	3	12
Countries represented (organisers)	Australia, Ethiopia, UK	Ethiopia, UK	Ethiopia, UK	Rwanda, UK	UK	Kenya, Zambia	Kenya, Zambia	4
Number of regional trainers	0	18	17	16	0	1	1	51
Number of countries represented (trainers)	Trainers were ACT faculty	10	10	7	1	3	3	18
Names of countries represented (regional trainers)		Ethiopia, Ghana, Kenya, Liberia, Malawi, Mozambique, Rwanda, Tanzania, Zambia, Zimbabwe	Ethiopia, Cameroon, DRC, Ghana, Kenya, Malawi, Sierra Leone, South Africa, Zambia, Zimbabwe	Burundi, Niger, DRC, Senegal, Cameroon, Rwanda, Togo				18
Cadres of trainers: Surgeon	5 M	11 M	9 M	5 M	0	0	0	30
Physiotherapist	2F, 1 M	1F, 5 M	1F, 2 M	8 M	0	0	0	20
Medical doctor	0	0	0	1F, 1 M	0	0	0	2
Clinical officer	0	1 M	3 M	0	0	0	0	4
Nurse/other	0	1F	2 M	1 M	0	0	0	4
Number of local providers trained	20	17	18	21	12	12	13	113
Cadres of providers trained: Surgeon	3 M	3 M	0	1 M	2 M, 1F	0	0	10
Physiotherapist	1F, 7 M	1F, 3 M	1F, 9 M	3F, 9 M	2 M, 6F	1 M, 2F	2 M, 1F	48
Nurse	7F	4F, 5 M	2F, 1 M	2 M	1F	1 M	0	23
Doctor	2 M	1 M	1F, 4 M	4 M	0	0	1 M	13
Other	0	0	0	0	0	6 M, 2F	5 M, 4F	17

*M* male, *F* female

^a^Several trainers were involved with multiple trainings. The total is the number of individuals involved and does not count a person more than once

The following examples demonstrate the type of information collected in the formative research and how this information was used to inform decisions about the approach to the training message.

First, the need to develop a training of trainers was developed as part of the grant application. The need to model best practice when training was further raised in the interviews and TAG meetings. In response, the TTT was developed to build the foundation for best practice training techniques. On the basis of the content and the observed need to be closely supervised and assisted in practical sessions, a 1:4 trainer to participant model was adopted when delivering the BPC. Closer supervision of trainees was required given the increased emphasis on interactive learning and the inclusion of detailed practical sessions.

Second, the proposed 1 day ‘Advanced Provider Course’ that was identified in the needs analysis was modified to the 2 day ‘Advanced Non-Surgical Clubfoot Treatment Course’ over the pilots. Challenges with scope of content were raised in the September 2015, January 2016 and July 2016 pilots. Small group discussions and responses from questionnaires advised that trainees with a good level of skill in clubfoot management (Ponseti technique), who are actively receiving mentoring and supervision, require an environment in which to refresh understanding and skills in basic clubfoot management and to delve deeper into how and why the technique works, in addition to sharing tips and advice through discussion of complicated cases. Consequently, it was agreed that changes to the APC were needed. A low-cost pilot in the UK was undertaken in December 2016, ahead of the pilot in Kenya in January 2017, to maximise the opportunity for success. The one-day advanced course was therefore developed with the intentional focus on non-surgical intervention. It was extended to 2 days to allow adequate time for discussion, reflection, practice and development of practical skills, based on pilot course feedback by trainers and participants. It was noted that a further course on surgical management is required.

### Knowledge and skills gained

Fifty-one regional trainers and 113 national clubfoot treatment providers (Ethiopia, Rwanda and Kenya) were trained through the course development. Results of a knowledge MCQ and confidence matrix completed pre- and post-training by local providers are demonstrated in Table 2.

**Table 2:** Knowledge and confidence of participants before and after training

	Pre-course mean (95%CI)	Post-course mean (95%CI)
BPC participants		
MCQ	59% (53–65)	80% (74–85)
Confidence	57% (50–64)	89% (86–92)
APC participants		
MCQ	59% (49–68)	73% (63–82)
Confidence	85% (79–92)	95% (92–98)

Correct multiple choice questionnaire (MCQ) answers increased from 59 to 80%, (BPC participants) and 59 to 73%, (APC participants) after training. Self-reported confidence increased from 57 to 89% (BPC participants) and 85 to 95% (APC participants) (Table 2).

## Discussion

This study outlines the formative research that informed the design of a training course for clubfoot providers in resource constrained settings. A two-day training course for novice clubfoot providers (Ponseti method) and two-day training course for advanced clubfoot providers were developed. Decisions were made using a data driven approach with a comprehensive contextual understanding, and involved key stakeholders. The ACT project delivered consensus on the content and quality of training material through partnership with technical advisory groups, regional trainers and local participants. The local organising teams, who were not involved in the training design, provided insight on practical issues and it is likely that their involvement will increase sustainability. In-depth needs analysis, interviews, surveys, opinion polls, group discussions with experts, consensus meetings, piloting the materials and observation allowed the development of a training course that aligned with regional priorities.

### Strengths

From the beginning of the ACT project there was agreement on the focus of the training course. The partnership development plan ensured commitment to health system partnerships and partnerships in practice. The flexibility in design of the course allowed the decision-making process to be iterative and the training materials to be modified as new information emerged. For example, the process allowed the development of two courses and identification that a further course focusing on surgical management was required, and the APC was expanded from a one-day to a two-day course in order to meet training needs as identified by contextual understanding, the training experts and participants. In addition, pooled resources (human, institutional and financial) contributed to the success of the training course design and the constant dialogue between colleagues, experts and stakeholders with different backgrounds facilitated exposure to different views and approaches [25, 26]. Stakeholder meetings, webinars and pilot trainings were used as arenas for decision-making on consensus issues and the various mediums were useful to build regional ownership.

### Limitations

A limitation of the training course is the absence of a pass/fail competency [27]. Instead, a skills checklist (Additional file 5) was developed to allow follow-up mentoring to identify areas of strength and weakness in novice providers. The skills checklist allows a skill gap analysis and follow up in supervision clinics [16]. Consensus was that the course would provide the initial foundation for the delivery of clubfoot treatment and further mentorship is required; as such a competency pass/fail was deliberately avoided. Seventy-three percent (19/26) of the experts invited for an interview and 68 % (68/100) of international trainers invited to complete the survey did not participate. The survey participants were also self-selected and selection bias in the development of the training curriculum cannot be ruled out. With regards translation, the forward translation was undertaken by a health professional, however the expert panel participating in the backward translation consisted of the regional francophone trainers. This research is limited to the description of how a training course was designed and the collaborative process undertaken, and intentionally does not evaluate the long-term outcomes or impact of the training course. The authors were closely involved in the implementation of the ACT project and in the development of the manuscript, which may lead to researcher bias in the results of this study.

### Lessons learnt

1. Within the ACT collaboration, each partner had unique characteristics without which the partnership would not have be possible (e.g. human resources, technical expertise, funding ability, awareness of context); common shared values and interests among the partners were essential.
2. The rigor and focus of the Health Partnership Scheme grant brought partners together with a sense of purpose and common goals.
3. Minutes that outlined the understanding of proceedings and action points to be undertaken after each meeting allowed an organised approach to communication and accountability.
4. The management of expectations and activities was aided by clearly defined objectives for the training that were regularly assessed and modified when necessary.
5. The constant evaluation of all aspects of the training materials, and their potential limitations, was promoted to inform the design and development of the training course.
6. The use of a variety of methods to deliver the training messages (such as demonstration, pair work, practical opportunities) was important to help select the anticipated behaviour changes.

The work we describe took 2 years to develop and will require further evaluation of regional implementation and impact.

### Challenges

The creation of partnerships between non-governmental organisations (NGOs) that are often in competition for funding, combined with avoiding a culture of individualism, took leadership and patience on all sides. Establishing consensus among multiple stakeholders was one of the most difficult and time-intensive steps, and required negotiation skills as well as a shared commitment to the ultimate goal. Meetings with international and regional stakeholders were limited by time as participation was on a volunteer basis. Practically, different time-zones and inconsistent internet access required web meetings to have thorough advanced planning. Barriers to volunteering with the ACT project faculty included difficulty in National Health Service staff taking time away from the UK, as observed in other projects [28].

### Main findings as related to previous literature

The importance of formative research for the design of successful interventions extends from the provision of neonatal care in rural Ghana [17], to hygiene promotion programmes [29] and programmes to reduce the treatment gap for mental health disorders in low resource settings [30]. Principles of good collaboration have been proposed to include (i) development across a diverse group of stakeholders, (ii) establishment of a community of practice, (iii) strengthened links between the project, communities, and ministries of health, and (iv) enhanced mutual respect for different cultures and contexts [14]. However, while interventions regularly include various forms of training, there are few published examples of the design process undertaken to create the training.

### Implications

Major gaps in evidence are highlighted through this research. There is little information available on the formation of training courses for health care workers in resource constrained settings. The accurate identification of processes that assist and facilitate learning and application of new skills within appropriate contexts is required. Further evaluation of the interaction between knowledge and competence is needed. In addition, evaluation of how different components of a training programme are understood by different cadres is required, as is the carry-over of skills after the training course.

### Recommendations for implementation of the training course

1. Training with practice-based learning requires direct supervision and multiple opportunities for the clubfoot providers to demonstrate competency in practice over time. In this example, the training is designed to occur in a 1 to 4 (trainer to provider) model to allow providers adequate participation and supervised practice in assessment and treatment.
2. Supportive supervision and mentoring ‘on the job’ is needed to meaningfully build on initial skills gained in training.
3. The sharing of knowledge of existing curricula, programmes and systems will increase opportunities globally to build regional capacity and increase access to interdisciplinary services.
4. Innovative ways to address the potentially limited access to clubfoot models or equipment suggested in this training warrants further research (e.g. through the use of mobile health tools).
5. National investments in provider training and in supporting the health system are required for scaling up and sustaining clubfoot treatment.

## Conclusion

Formative research with mixed methods (both quantitative and qualitative) was essential for the development of the training courses. Consensus meetings were central to the harmonisation of aims and goals of the ACT project. A broad spectrum of multidisciplinary stakeholders, beyond those with clinical expertise, shaped the success of the training project through a shared vision and mutual accountability. Knowledge increased in both novice and advanced participants after training. Self-reported confidence increased on all measures tested after participating in the training. The process data from this study provide useful information to assist planning of medical training programmes and may serve as a model for the development of other courses.

## Additional files

Additional file 1: Survey of current training practices. Includes questions and topic guides for survey that aimed to understand practical issues, current knowledge and skills gaps, and follow up mentoring requirements. (DOCX 21 kb)

Additional file 2: Considerations for equipment and location. A list of material inputs that was created over the pilot trainings to assist the planning and organisation of future training courses. (DOCX 76 kb)

Additional file 3: Example of the basic provider course MCQ. The pilot tested single best answer multiple-choice questionnaire. (DOCX 25 kb)

Additional file 4: Finalised training course components. Outlines the components of the training courses. (DOCX 15 kb)

Additional file 5: Skills checklist. The checklist developed to allow follow-up mentoring to identify areas of strength and weakness in novice providers. (DOCX 17 kb)

Additional file 6: Acknowledgements. Acknowledgements of the multi-disciplinary team involved in the ACT project. (DOCX 15 kb)

## Abbreviations

ACT: Africa Clubfoot Training
APC: Advanced provider course
BPC: Basic provider course
GCI: Global clubfoot initiative
MCQ: Multiple choice questionnaire
NDORMS: Nuffield Department of Orthopaedics, Rheumatology and Musculoskeletal Sciences
NGO: Non-Government Organisation
TAG: Technical advisory group
THET: Tropical Health Education Trust
ToC: Theory of change
TTT: Train the trainer
WHO: World Health Organisation
