# Supplementary material for: The development of a training course for clubfoot treatment in Africa: learning points for course development
Source: BMC Med Educ. 2018 Jul 13;18:163. doi: 10.1186/s12909-018-1269-0 (PMC6044045; doi:10.1186/s12909-018-1269-0)
Supplement: Supplementary file 1 — Survey of current training practices. Includes questions and topic guides for survey that aimed to understand practical issues, current knowledge and skills gaps, and follow up mentoring requirements. (DOCX 21 kb) [file 12909_2018_1269_MOESM1_ESM.docx]

**Additional File 1: Survey on current training practices**

**1. Please tell us about your experiences of conducting Ponseti training...**

How many Ponseti training courses have you been a trainer on in the past?

What level of Ponseti courses have you taught? (beginner/advanced courses, or both)

Which countries were the Ponseti courses held in?

**2. When delivering Ponseti courses, what training materials have you used?**

GCI training materials

Your own training materials

Other training materials (Please specify below)

Other

**3. If you have used the GCI training materials please comment on how they could be improved.**

**4. In your experiences of training, what is the best format to deliver a Ponseti training course:**

1-part course: Teach all theoretical and practical aspects at once

2-part course: Start with a preliminary ‘beginners course’ followed by an ‘advanced’ or ‘refresher’ course some time later.

Comments

**5. If you were to deliver a 2-part course starting with a preliminary ‘beginners course’ followed by an ‘advanced’ or ‘refresher’ course what modules should be included in each course:**

Please suggest any additional modules that should be included

**6. What ‘red flags’ or potential complications of treatment would you make course participants aware of during a beginner’s course? (tick all that apply)**

Atypical or complex appearance and features

Cast slips

Casting sores

Under-correction of the foot

Over-correction of the foot

Casting errors such as pronating or pushing the foot into dorsiflexion

Relapse of foot deformity

Patient dropping out of treatment

Others (please list below in comments box)

Other (please specify)

**7. What hands-on practice elements should be included in a Ponseti course? (tick all that apply)**

Practice manipulation on clubfoot skeleton models

Manipulation and casting using rubber foot models

Manipulation and casting on patients

Brace fitting on patients

Pirani scoring on patients

Any other practical exercises you would recommend? (please list in the comments box below)

Other (please specify)

**8. Ponseti course practicals...**

For practical exercises, what is the ideal number of trainees per trainer?

How many children should each person practice casting on, ideally?

**9. Is a 2-3 day Ponseti course with theoretical and practical elements sufficient for participants to learn how to apply the Ponseti technique effectively?**

Yes/No

If not, what additional elements would you suggest?

**10. Do you have any other comments or suggestions on the best ways to deliver training in the Ponseti technique?**
