# Supplementary material for: The development of a training course for clubfoot treatment in Africa: learning points for course development
Source: BMC Med Educ. 2018 Jul 13;18:163. doi: 10.1186/s12909-018-1269-0 (PMC6044045; doi:10.1186/s12909-018-1269-0)
Supplement: Supplementary file 2 — Considerations for equipment and location. A list of material inputs that was created over the pilot trainings to assist the planning and organisation of future training courses. (DOCX 76 kb) [file 12909_2018_1269_MOESM2_ESM.docx]

**Additional File 2: Considerations for equipment and location**

1. Choice of course location and venue : consider – regional versus central, cost, space for both lectures and practicals, noise, ease of catering, accommodation nearby, experts
2. Create an agenda that is realistic, allowing for set-up time each day, breaks, travel times, practical sessions, debriefing
3. Organise equipment – projector, laptop, power blocks, flipcharts, buckets, plinths, scissors, plaster cutters
4. Organise consumables – plaster, padding, braces, gloves, tenotomy supplies
5. Organise resources – electronic copy of slides, manuals, handouts, blank patient records, certificates of attendance
6. Set up the day before the course – room set-up, check equipment is working, organize handouts / manuals / stationery, ensure course materials are on laptop, consider layout / equipment for different sessions
7. Consider publicity / media for the course
8. Organise accommodation, catering, and transport
9. Arrange a faculty / team meeting before the course – allocate topics / sessions / roles:
   1. Course leader
   2. Timekeeping and liaise with catering team
   3. Allocated instructor for each session
   4. Preparation for practical sessions including group sizes and room layout (before course and on the day)
   5. Technical support (computers)
   6. Invitations, registration, and paperwork
   7. Giving each other feedback
   8. Coordinating patients and families
   9. Set-up and set-down
