# Supplementary material for: The development of a training course for clubfoot treatment in Africa: learning points for course development
Source: BMC Med Educ. 2018 Jul 13;18:163. doi: 10.1186/s12909-018-1269-0 (PMC6044045; doi:10.1186/s12909-018-1269-0)
Supplement: Supplementary file 3 — Example of the basic provider course MCQ. The pilot tested single best answer multiple-choice questionnaire. (DOCX 25 kb) [file 12909_2018_1269_MOESM3_ESM.docx]

**Additional File 3: Example of the Basic Provider Course MCQ**

**Basic Ponseti Provider Course – Multiple Choice Questionnaire (MCQ)**

There are 12 questions, with 1 correct answer for each question. There is no negative marking. Please answer every question, and guess the correct answer if you are not sure.

1. Which of the following is not a clinical component of clubfoot?

1. Equinus
2. Abductus
3. Varus
4. Cavus

2. Which of the following is not a goal of the Ponseti Method?

1. A functional foot
2. A plantargrade foot
3. To use modified shoes
4. A painfree foot

3. According to the Pirani score, when should a tenotomy be performed?

1. When the midfoot score is more than 1
2. When the midfoot score is 0
3. When the hindfoot score is 0
4. When the lateral head of talus score is 1

4. Which of the following is not assessed in the Pirani score?

1. Posterior Crease
2. Empty Heel
3. Degrees of Abduction
4. Medial Crease

5. Which combination of elements are required by the Ponseti method to successfully manage a clubfoot?

1. Manipulation and foot abduction brace
2. Manipulation, casting, Achilles tendon tenotomy and foot abduction brace
3. Casting and Achilles tendon tenotomy
4. Manipulation, casting and Achilles tendon tenotomy

6. What is the Pirani score NOT helpful for?

1. Using a common language to discuss patients with other clubfoot providers
2. Informing your next treatment
3. Deciding when to discharge your patient
4. Monitoring correction of deformity

7. Which is NOT required for successful Ponseti casting?

1. Discussing the care of the cast with parents
2. An aim for the cast
3. Two people to cast
4. Manipulation of the foot after casting

8. Why is moulding of the cast important?

1. To make the cast look nice for the parents
2. To keep the manipulated position of the foot
3. To give you something to do as the cast dries
4. To strengthen the cast so that the child can walk on it

9. What position does the first Ponseti cast maintain?

1. Dorsiflexion of the ankle
2. Abduction
3. Pronation
4. Supination

10. What deformity of clubfoot does the first Ponseti cast correct?

1. Adductus
2. Cavus
3. Equinus
4. Varus

11. The ‘lateral head’ of which bone is the fulcrum for manipulation with the Ponseti method?

1. Calcaneum
2. Lateral Malleolus
3. Navicular
4. Talus

12. What is INCORRECT in your assessment of brace quality?

The brace should have:

1. Shoes attached to bar at 50^o^ abduction
2. Dorsiflexion of 10^o^-15^o^
3. A well rounded heel cup
4. An inspection hole on the medial side
