# Supplementary material for: The development of a training course for clubfoot treatment in Africa: learning points for course development
Source: BMC Med Educ. 2018 Jul 13;18:163. doi: 10.1186/s12909-018-1269-0 (PMC6044045; doi:10.1186/s12909-018-1269-0)
Supplement: Supplementary file 4 — Finalised training course components. Outlines the components of the training courses. (DOCX 15 kb) [file 12909_2018_1269_MOESM4_ESM.docx]

**Additional File 4: Finalised training course components**

| **Course** | **Components** |
| --- | --- |
| Basic Provider Course | Introduction to clubfoot and Ponseti management  Anatomy and definitions  Pirani score  Clubfoot deformity and Ponseti method of manipulation  Practical session with rubber and skeletal models  Applying Ponseti casts  Demonstration of Pirani score and casting on 2 children  Practical session with casting on rubber models  Tenotomy  Bracing and relapse  Practical session on assessment  Manipulation and casting on patients  Practical session on brace fitting and tenotomy  When to stop and re-think treatment |
| Advanced Non-surgical Clubfoot Treatment Course | Review of clubfoot management (Ponseti Method)  Why the method works (alternative hand holds and kinematic coupling)  Practical exercises with skeletal models  Common errors  Atypical cases including video and case study  Management of recurrence  Treating older children  Syndromic cases  Practical exercises with casting on rubber models  Group assessment of patients and formulation of treatment plan  Demonstration of treatment on patients  Clinic and treatment quality  Parent education and adherence |
