# Supplementary material for: The development of a training course for clubfoot treatment in Africa: learning points for course development
Source: BMC Med Educ. 2018 Jul 13;18:163. doi: 10.1186/s12909-018-1269-0 (PMC6044045; doi:10.1186/s12909-018-1269-0)
Supplement: Supplementary file 5 — Skills checklist. The checklist developed to allow follow-up mentoring to identify areas of strength and weakness in novice providers. (DOCX 17 kb) [file 12909_2018_1269_MOESM5_ESM.docx]

**Additional File 5: Skills checklist**

| **Training skills check** | **Skills demonstrated under supervision** | **Observed** | | **Not in phase of treatment** |
| --- | --- | --- | --- | --- |
|  |  | **Yes** | **No** |  |
| **Discuss Ponseti treatment with parents** | Uses demonstration or descriptions to communicate to parents |  |  |  |
|  | Discusses the plan for treatment |  |  |  |
|  | Asks if the parents have questions |  |  |  |
| **Assessment Pirani score** | Completes entire Pirani score form including date and initials |  |  |  |
|  | Interprets and analyses findings to establish treatment plan |  |  |  |
| **Manipulation of child’s foot** | Good position of child and parent |  |  |  |
|  | Identifies lateral head of talus, correct handhold |  |  |  |
|  | Manipulates foot into accurate position to correct deformity |  |  |  |
|  | Uses gentle handling, 'soft hands' |  |  |  |
| **Casting of child's foot** | Cast over toes |  |  |  |
|  | Lower half of cast applied first |  |  |  |
|  | Cast up to groin and gluteal fold |  |  |  |
|  | Moulds cast until dry |  |  |  |
|  | Knee at 90 degrees |  |  |  |
|  | Toes visible in casts |  |  |  |
|  | Regular talking with moulder |  |  |  |
| **Application of brace** | Correct measurement of foot and selection of brace |  |  |  |
|  | Looks at heel cup to ensure heel is in brace correctly |  |  |  |
|  | Accurately conveys information regarding relapse |  |  |  |
|  | Makes appointment for review of brace |  |  |  |
