# Supplementary material for: The development of a training course for clubfoot treatment in Africa: learning points for course development
Source: BMC Med Educ. 2018 Jul 13;18:163. doi: 10.1186/s12909-018-1269-0 (PMC6044045; doi:10.1186/s12909-018-1269-0)
Supplement: Supplementary file 6 — Acknowledgements. Acknowledgements of the multi-disciplinary team involved in the ACT project. (DOCX 15 kb) [file 12909_2018_1269_MOESM6_ESM.docx]

**Additional File 6: Acknowledgements**

The multi-disciplinary ACT team involved in assembling, writing, piloting, reviewing and rewriting the material includes:

Endashaw Abera, Kinfe Araya, William Guy Atherton, Birhanu Ayana, Ryan Bathurst, Rachel Buckingham, Alexis Buunaaim, John Cashman, Christopher Carter, Augustine B. Chiewolo, Sandram Chimangeni, Naomi Davis, Marieke Dreise, , Roderick Duncan, Mesfin Etsub, Jennifer Everhart, Solomon Fasika Demissie, Gregory Firth, Rick Gardner, Prosper Guo-Moh, Ben Gwilliam, Lin Habimana, Soeur Odette Habimana, Jean Claude Habyarimana, Linda Hansen, Moussa Moise Henri Martin, Alison Hulme, Nathaniel Sallu Kargbo, Stephen Kariuki, Christian Katembo Kamavu, Kakule Katenge Joseph, Pascal Kayishema, Simplice Kighoma Vuhaka, Charles V. Alain Kinkpé, Peter Klungsøyr, Joseph Korpisah, Koffi Kouwekou, Giorgio Lastroni, Chris Lavy, Grace Le, Benjamin Lwayivweka Ngahangondi, Chouchou Safi Matsoro, Deborah McMillan, Samuel Maina, Paul Mang’oli, Stephen Mannion, Osman Ibrahim Mohamud, Karen Moss, Ilho Moyo, Debra Mudariki, Ibrahim Issaka Niandou, Isidor Ngayomela, Marie-Caroline Nogaro, Tim Nunn, Henry Ndasi, Jean-François Negrini, André Georges Nguene Nyemb, Emmanuel Nsengiyumva, Isaac Otieno, Rosalind Owen, Safalao Phalira, Norgrove Penny, Rebecca Radcliffe, Scott Reichenbach, Sampson Sarpong-Peprah, Prem Saggurthi, Peter Matthias Schmauch, Jana Shih, Kamwanda Sililo, Tracey Smythe, Michiel Steenbeek, Unisa A Tarawallie, Tim Theologis, Joseph Theuri, Tewodros Tilahun, Tchaa Hodabalo Towoezim, Michael Uglow, Esperance Uwizeye, Bernard Uzabakiriho, Andrew Wainwright, Situmbeko Wambulawae, Denise Watson and Kagnew Wubishet.

In developing these materials we acknowledge the foundational work of many individuals over the years that the Ponseti technique has been practiced in Africa. We are very grateful for material shared by many in the group above, and we also thank the following for a combination of expertise, advice, inspiration and photographs: CBM, Winfried Danke, Fred Dietz, Matthew Dobbs, Marieke Dreise, Bryce Flurie, Vikas Gupta, John Herzenberg, Iris Lohan, A F Lourenco, Colin MacFarlane, Jennifer McCahill, José Morcuende, Vince Mosca, Monica Noguiera, Norgrove Penny, Shafique Pirani, Ignacio Ponseti, David Scher, Marc Sinclair, David Spiegel, Lynne Staheli, Michiel Steenbeek, UK Clubfoot Consensus Group, Miraclefeet and Walk for Life Bangladesh.
